# Causes of Decline in the University of Edinburgh

**Published:** 1834-04-01

**Authors:** 


### An Examination into the Causes of the Declining Reputation of the Medical Faculty of the University of Edinburgh, &c. &c.

Review

This pamphlet has created some sensation in the medical world, especially amongst those members of the profession who have received their education in the northern metropolis, or graduated there. The number of these is not by any means small; and when we look around upon those practising, with so much credit to themselves, in Modern Babylon alone, we confess that it gives us much pain to hear the above fact asserted, and still more to learn the principal causes as stated in this Examination. "The days of the Blacks, the Cullens, Gregories, and Monros, are no more, and the fame of the Northern Athens has departed with them." We who have seen the theatres of some of them daily filled to their highest benches with students listening, with unceasing interest, to their Professors' prelections, the termination to which was, their noisy, though heart-cheering applause, are tempted to exclaim, on re-entering the collegiate portals—"The friends of my youth where are they? and the echo answers—where are they?"

That the Edinburgh University has declined in reputation owing to some cause or other within the last few years is the general opinion, and the author of the above pamphlet has entered upon the task of proving to the profession and to the public, the direct and latent causes of this falling off. Three principal ones are stated, as follows:—

1st. The election of persons, not properly qualified for the office, to the different professorships.

2dly. The students being overwhelmed by compulsory attendance on unnecessary lectures.

3dly. The character of the examinations in the University for degrees being bad, if not disgraceful.

We apprehend that many other causes exist to account for the gradual diminution of the University students, besides those brought forward by the author, and we cannot altogether coincide with him even in adopting these as facts, and therefore must dissent from many of his deductions.

That the elections of some of the professors to chairs in the University of Edinburgh have been owing to political interests, family connexions, and other unfair means, we are not at all disposed to deny. The qualifications of the candidates, in these cases, have weighed as dust in the balance against their more powerful opponents, self-interest and favouritism. An exposition of the curious machinery set in motion at these epochs, and of the *oil* used to keep its wheels in revolution, would be amusing, though at the same time it would give us very unfavourable ideas of some of the principal worthies connected with the affair. The professorial chair of physic, for example, so brilliant in the days of Cullen and Gregory, has, since the death of the latter revered teacher, been a dead letter; not perhaps owing to any lack of knowledge in its present Professor, but from various impediments which might have prevented even a profound philosopher from gaining any increased reputation as a lecturer. Nor can the Glasgow college boast of being more fortunate in this respect, owing to the secret influence exerted, 400 miles from the spot, to secure the chair for a favoured scion of an English University! In selecting an individual to fill this, (generally considered as the principal Professorship of a medical college) great care should, no doubt, have been devoted to the choice of one who, by his extensive knowledge and practice, should have been known to all—whose aptitude for investigation in this important branch of study—and whose unwearied diligence, in preparing himself for the diffusion of its result, to his audience, might fairly compete with all rivals in maintaining the celebrity of the chair. Let us hope, however, that a recent appointment in the Glasgow University, gives signs of better times henceforward; and that the system of nepotism and court influence is on the wane. A few more such professors will make it a formidable rival to Edinburgh.

In following the author, we find him objecting to several of the appointments to the University chairs, either owing to the non-ability of the individuals filling them, or, on account of the absence of necessity for the endowment of such chairs at all. *Pathology*, for instance, is considered as unworthy of such a distinction, as it is taught by all anatomical lecturers. Every one will, however, recollect how very scantily it is treated on, in these courses, and indeed how few opportunities, comparatively, arise for good demonstrations in this department of the lectures. Much time is required for any thing like a fair study of the different morbid structures, and we may instance the late work which has appeared on the structure of the liver only, by Mr. Kiernan, which is the result of two years' intense application. The University of London has lately thought it of sufficient importance to require a separate professorship, that able pathologist, Mr. Carswell, now holding the appointment. When, indeed, we consider the immense practical importance very properly attached to an intimate knowledge of the morbid condition and various appearances of diseased tissues, we cannot justly blame a medical corporation for giving to their students every possible facility of making themselves thoroughly acquainted with the subject.

The appointment to the chair of clinical surgery is strongly animadverted on by the author, both on account of the nature of the transfer by sale, and of the youth and inexperience of the Professor. As relates to the former, we must decidedly attach much blame to the governing body, for permitting such a transaction to occur; the old Professor who has (according to the author) received a large income from this appointment for many years, could in no way be entitled to any remuneration for quitting a chair of which he was no longer able to perform the duties. That the change, however, has proved, in an immeasurable degree, beneficial to the students, we suspect will be universally acknowledged by them, unless these lectures were latterly conducted in a far different manner to what they were some 13 or 14 years since. The founder and assiduous superintendant of an excellent clinical hospital, the reports from which have, at various times, afforded much useful information to the profession; the experienced anatomist who has been engaged in the arduous occupation of lecturing for the last 12 years, we cannot conceive to be ill-qualified, either from youth or inexperience, for an office requiring much activity both of mind and body in selecting and following up the different interesting cases which may occur in the wards of the hospital. The compulsory attendance on these lectures seems, however, to our author, a great hardship on graduates of medicine, who should not be required to possess a *thorough* knowledge of this branch of instruction!! Before quitting this subject, we put it to the author, in reference to the appointment, whether it would have been either fair or proper to have conjoined the two professorships of surgery to the College of Surgeons, and that of clinical surgery to the University, in one and the same person?

Again, we are not at all surprised at the University of Edinburgh having found it requisite to raise the standard of qualifications for their degree; and, in so doing, we consider that it has acted wisely and prudently, in order to keep pace with the advance of medical science within these last few years. Let us look back, for instance, at the manner in which anatomy was taught 15 or 16 years since, and then consider, how much more scientific a method the French and German anatomists have forced us to adopt, as a more free intercourse with them has enabled us to profit by a closer intimacy with their works. Scotch degrees, we are aware, have been, in some mouths, a bye-word, owing to the lax discipline and scanty qualifications required by St. Andrew's and Aberdeen; and should Edinburgh therefore be arraigned for endeavouring to keep her good name, and avoiding all such reproach, and for causing her alumni to be respected by the amount and quality of their acquirements? The Apothecaries' Hall in London has been lauded by all, for the self-same advance, in contrast with the Royal College of Physicians. We must disagree also with the author, in the opinions he expresses as to the source of information being an entirely unnecessary consideration, on a candidate presenting himself for a degree in any University. We confess that we dislike the plan of a Senatus Academicus sitting ready to examine all persons who may arrive, per mail or steam, for that purpose, and departing next day with the summi honores in their pockets. *Some part* of the probationary studies should be passed within her walls, in order to prevent her becoming a mere shop for the sale of degrees. That the teachers should not themselves be examinators, we are prepared fully to admit. This evil has been long felt in our College of Surgeons, and we sincerely trust that the legislature will, amongst other abuses, correct this glaring one. Examining masters for the purpose, similar to the Oxford and Cambridge officers, would be more accordant with propriety and justice.

The lengthened attendance on hospital practice is a point we agree perfectly with the faculty in thinking an improvement; and on this question, therefore, we are at complete issue with the author. If any one branch of our preliminary education, as good practical physicians, is of supreme importance, it is the observation and constant attendance at the bedside of patients in an hospital; and the clinical wards of the Edinburgh Infirmary are, from the excellent method of their arrangement and routine practice, an example to the London hospitals, and certainly beneficial, in an exemplary degree, to those students who follow diligently the opportunities afforded them. *Medical jurisprudence* is another branch of medical study, which the author objects to, as requiring a separate chair. The manner in which it is taught by the professors of chemistry, midwifery, and materia medica, although sufficient in the eyes of the author, we hold to be utterly incompetent and unsatisfactory for the education of a physician. Let us remember in what an unenviable position some of our medical brethren have been placed in courts of justice, continually, from a want of knowledge on this subject. It is unfortunate, likewise, for the author's position, that it is now *required* at the Apothecaries' Hall, and is taught even in the London hospitals.

The quotation of Oxford, as any authority on medical legislation, we conceive to be peculiarly unfortunate. It is true that that university requires no imperative curriculum of study, but that the candidate is required to devote three years to the study of medicine elsewhere; and now let us ask to which university do they then generally bend their steps; and where do they gain the knowledge which is to fit them for their after examinations? We apprehend that no worse authority than Oxford could have been adduced; for it is notorious that the graduates, glorying in the degree of that university or of Cambridge, *do* obtain *all* the information, (which is worth anything to the public,) in their capacity of physician, ELSEWHERE!!

The system of examination in the Edinburgh University the author reprehends as disgraceful. That many blockheads have there obtained degrees we will ourselves at once confess; but we will, in reply, ask the author to point out any learned body in this or in other kingdoms, and say if he cannot instance some one or more individuals who form component parts of those societies, and who are not most fully entitled to the same appellation? Where examinations are strict and proper, still chance may direct the questions in them to those identical topics, of which *alone* perhaps the candidate has a knowledge. We have ourselves known instances of men who went direct from the book to this dreadful tribunal, and to their inexpressible gratification, the first questions were on the subjects of their last read page, the answers being so remarkably satisfactory to the examiners that the after parts of their examination were lightly passed over, owing to the thorough knowledge evinced on all the subjects for the first half hour. Instances of this are of frequent occurrence at our universities, and cannot easily be avoided. As far as we are able to decide, from experience, we should say that the examinations for the degree of Doctor Medecinæ in the University of Edinburgh, are, in comparison with three others of which we have personal knowledge, the most difficult, and though of course susceptible of some improvement still, are decidedly not inferior to those of any medical college in Great Britain. A very fair quota of aspirants are usually sent back to undergo further examination, proving that it is not the love of filthy lucre alone which actuates the minds of the professors.*

In a former part of this critique we mentioned that the author had not taken into consideration some causes which might strongly affect the interests of the Edinburgh University, and account, in part, for the decrease of its pupils from the sister kingdoms. He has, however, we find, in again looking over the "Examination," slightly touched upon these topics; but attributes little influence to them. He must recollect, however, that the schools of London have, of late, become both more numerous and attractive than formerly. Hospitals which, a few years since, had no schools attached to them, have considered it their interest to found them. The University of London and King's College have operated against Edinburgh still more; and should a faculty in London be invested with the power of conferring degrees, it will altogether prevent the egress of candidates for a degree from England. Those students too, who, having finished their probationary studies in London, formerly visited Edinburgh to complete their education, now prefer Paris, on many accounts, for that purpose.

One word more as to the examinations in Edinburgh, which the author finds fault with, as rendering concealment of the rejection of any candidate impossible. *So much the better.* If they were conducted altogether in public, it would have the greatest possible benefit in putting a stop to that negligence and dissipation of the students so justly remarked on by the author. "They order this matter better in France." As to the power of the Edinburgh University in granting degrees, we apprehend that the author must be in fault in denying it—if he be correct, not a day should be lost in applying to Parliament on the subject.

In conclusion, we think that the author, though taking different views on many subjects from ourselves, has handled them fairly, and has adduced many striking facts in his pamphlet, sufficient to put the new magistracy on their guard in future elections, if they possess the wish of upholding the fair fame of their colleges, especially when St. Andrew's and Aberdeen are at last endeavouring to rival them by awaking from the sluggish apathy which has so long been both their bane and disgrace. The author appears to have the interest of the university at heart, as well as that of the private lecturers, (we presume of course that he is of the latter class himself,) and we should ourselves recommend to the faculty of the former institution, to use no other means of suppressing this very useful class in the profession than by shewing (from the excellence of their own lectures) that they are unnecessary.

---

* We do not, however, consider the mode of examination in Edinburgh, or in any of our colleges, as by any means the most proper tests of medical acquirements. Nothing but a public examination, with the subjects before them, can ever prevent ignorance from escaping, by means of cramming, and foolish or improper questions being put by superannuated, prejudiced, or pedantic examiners.—*Ed.*

